# Relevance of the NR4A sub-family of nuclear orphan receptors in trophoblastic BeWo cell differentiation

**DOI:** 10.1186/s11658-017-0046-0

**Published:** 2017-08-09

**Authors:** Sudha Saryu Malhotra, Satish Kumar Gupta

**Affiliations:** Reproductive Cell Biology Laboratory, National Institute of Immunology, Aruna Asaf Ali Marg, New Delhi, -110 067 India

**Keywords:** Nor-1, Nurr-1, Nur-77, Trophoblast differentiation, hCG, GnRH

## Abstract

Nur-77, a member of the NR4A sub-family of nuclear orphan receptors, is downregulated in the placentae of pre-eclamptic women. Here, we investigate the relevance of Nor-1, Nurr-1 and Nur-77 in trophoblastic cell differentiation. Their transcript levels were found to be significantly upregulated in BeWo cells treated with forskolin. The maximum increase was observed after 2 h, with a second peak in the expression levels after 48 h. The expression of NR4A sub-family members was also found to be upregulated in BeWo cells after treatment with hCG and GnRH. A similar significant increase was observed at the respective protein levels after 2 and 48 h of treatment with forskolin, hCG or GnRH. Silencing Nor-1, Nurr-1 or Nur-77 individually did not show any effect on forskolin-, hCG- and/or GnRH-mediated BeWo cell fusion and/or hCG secretion. After silencing any one member of the NR4A sub-family, an increase in the transcript levels of the other sub-family members was observed, indicating a compensatory effect due to their functional redundancy. Simultaneously silencing all three NR4A sub-family members significantly downregulated forskolin- and hCG-mediated BeWo cell fusion and/or hCG secretion. However, a considerable amount of cell death occurred after forskolin or hCG treatment as compared to the control siRNA-transfected cells. These results suggest that the NR4A sub-family of nuclear orphan receptors has a role in trophoblastic cell differentiation.

## Background

The multinucleated syncytiotrophoblast formation that occurs during placental development is a consequence of cell–cell fusion. The syncytiotrophoblast is involved in fetal–maternal nutrient exchange and the synthesis of steroid and peptide hormones, such as progesterone and human chorionic gonadotropin (hCG), which play a crucial role in pregnancy maintenance and fetal development.

A significant proportion of pregnancy-related complications, including pre-eclampsia, intrauterine growth restriction (IUGR) and spontaneous abortions are a consequence of impaired syncytialization [1–3]. Several autocrine or paracrine factors, including hormones, cytokines and growth factors, have been implicated in regulating cytotrophoblast differentiation to form syncytiotrophoblast [4]. hCG, a peptide hormone that is a marker of syncytiotrophoblast differentiation, has also been shown to play an important role in enhancing trophoblast fusion [5–7].

As syncytialization is an early differentiation event of the trophectoderm, understanding the molecular networks downstream of various inducers might give better insight into its regulatory processes. The BeWo choriocarcinoma cell line has been used as an established model to study trophoblast differentiation in vitro [8]. Unlike primary cytotrophoblast cultures, which undergo spontaneous fusion in the presence of fetal bovine serum, BeWo cells have a low spontaneous fusion potential. They can be induced to differentiate by various cyclic adenosine monophosphate (cAMP) analogues, like dibutyryl cAMP and cholera toxin, and by cAMP activators, like forskolin [9, 10].

The NR4A sub-family is among the most evolutionarily conserved within the nuclear receptor super-family. It has three known members: NR4A1, also called Nur-77 (neuron-derived clone 77); NR4A2, also called Nurr-1 (nuclear receptor-related protein-1); and NR4A3, also called Nor-1 (neuron-derived orphan receptor 1). In contrast to other nuclear receptors, the ligand-binding pocket in their structure is inaccessible, which is why they are also called nuclear orphan receptors [11, 12].

Unlike other ligand-activated nuclear receptors, the NR4As are transcription factors. Their activity is mainly regulated at the transcriptional level and in some instances, even by post-translational modifications and by direct or indirect interaction with several other co-regulatory factors. These co-factors are involved in a wide range of biological processes, induced by multiple stimuli [13, 14]. Kudo et al. [15] investigated the gene expression over time of BeWo cells induced to differentiate by exposure to forskolin. Studying the differentially expressed mRNAs at various time points yielded an elaborate picture highlighting important genes involved in cell adhesion, placental hormone synthesis, and fusion. Nor-1 was found to be the most upregulated gene, with an immediate early expression at 1 to 2 h [15]. Nur-77 expression was also detected in the villous and extravillous cytotrophoblast cells of the placenta, with its expression peaking during the first trimester. In another study, Nur-77 was found to be downregulated in the placentae of women suffering from pre-eclampsia [16].

In light of the existing literature, it is interesting to investigate the possibility of NR4A involvement in trophoblastic cell differentiation. The BeWo cell line was chosen as a model system. First, the expression profile of the NR4A members was observed in BeWo cells after treatment with forskolin. Then, a few physiological inducers of trophoblast differentiation, such as hCG and gonadotropin-releasing hormone (GnRH) were used [5, 17, 18]. Finally, siRNA-mediated silencing of all three members was carried out to discern their relevance to BeWo cell fusion.

## Methods

### Cell culture

BeWo choriocarcinoma cells were cultured in Ham’s F-12 medium (Sigma-Aldrich Inc.) enriched with 10% heat-inactivated fetal bovine serum (FBS; Life Technologies Corp.) supplemented with an antibiotic and antimycotic cocktail consisting of 100 units⁄ml penicillin, 100 μg⁄ml streptomycin and 0.25 μg⁄ml amphotericin B (Biological Industries, Kibbutz beit Haemek). They were maintained at 37 °C in a humidified atmosphere with 5% CO_2_. The cells were sub-cultured at around 70% confluence.

### Gene silencing by siRNA

BeWo cells (0.1 × 10^6^/well) were seeded in 6-well culture plates (Greiner Bio-One) in Ham’s F-12 medium with 10% FBS. At 80% confluence, cells were transfected with Nor-1, Nurr-1 or Nur-77 siRNA individually (sc-38,842, sc-36,111, sc-36,109; Santa Cruz Biotechnology Inc.) at an optimized concentration of 40 pmol using Lipofectamine 2000 (Life Technologies Corp.).

In another experiment, Nor-1, Nurr-1 and Nur-77 were silenced simultaneously: 30 pmol of each siRNA was mixed together in Opti-MEM to which Lipofectamine 2000 in Opti-MEM solution was then added. This mixture was added drop by drop over the cells in the 6-well plate.

After 4 to 6 h of incubation at 37 °C in a humidified atmosphere with 5% CO_2_, 1 ml of 20% FBS containing Ham’s F-12 medium was added to the cells. Next day, the media was replaced with Ham’s F-12 medium supplemented with 10% FBS and kept for 48 h. An appropriate silencing control with non-genomic siRNA was also included. After 48 h, the cells were trypsinized and viable cells were counted using the Trypan Blue exclusion test. Viable cells were further processed for the various experiments.

### In vitro differentiation of BeWo cells

Cells (0.3 × 10^5^⁄well) were grown on cover slips in 24-well culture plates (Greiner Bio-One) in Ham’s F-12 medium supplemented with 10% FBS. After 24 h, the cells were washed and starved for 4 h with Ham’s F-12 medium without FBS.

To induce differentiation, cells were further cultured for 48 h keeping appropriate vehicle control in serum-free Ham’s F-12 medium supplemented with 1 × ITS + 1 (Sigma-Aldrich Inc.) containing insulin (10 μg⁄ml), transferrin (5.5 μg⁄ml), selenium (0.005 μg⁄ml), linoleic acid (4.7 μg⁄ml), and bovine serum albumin (BSA; 500 μg⁄ml) with an optimized concentration of forskolin (25 μM), hCG (5 IU/ml) or GnRH (10 ng/ml; all three from Sigma-Aldrich Inc.).

After 48 h, the conditioned medium was harvested and the cells were fixed with chilled methanol for 5 min at 4 °C and processed for desmoplakin I + II staining. Slides were screened for immunofluorescence under a fluorescent phase contrast microscope (Nikon Instruments Inc.), and images were captured using Nikon Image Proplus software.

For each experimental group, fused cells were counted from 8 to 10 randomly selected microscopic fields. For each microscopic field, the number of nuclei in the syncytium and the total number of nuclei in that microscopic field were counted and their ratio was used to calculate the percentage of fusion. The results were represented as fold change in fusion with respect to their untreated controls.

### Quantification of hCG in the conditioned medium

The harvested conditioned medium was centrifuged at 3000 x g for 5 min to remove cell debris and stored at −20 °C until use. Solid-phase sandwich ELISA was performed to estimate hCG levels using a DRG β-hCG ELISA kit following the manufacturers’ instructions (DRG Instruments GmbH).

### Quantitative RT-PCR

BeWo cells (0.1 × 10^6^/well) were seeded in 6-well culture plates and cultured for 24 h. Cells were serum starved for 4 h before the addition of forskolin (25 μM), hCG (5 IU/ml) or GnRH (10 ng/ml) in Ham’s F-12 medium supplemented with 1 × ITS + 1 for different time intervals. Total RNA was isolated from cells using Tri reagent (Sigma-Aldrich Inc.) following the standard protocol employing chloroform–isopropanol–ethanol steps for purification. The RNA concentration in the samples was analyzed using a NanoDrop 3300 spectrophotometer (Thermo Scientific). The samples were also subjected to DNase I treatment at 37 °C for 15 min, as per the manufacturer’s instructions (Fermentas). The isolated RNA (5 μg) was used to prepare complementary DNA using a mix of random hexamers and oligo (dT) 18 primers, dNTP mixture, RT buffer and Maxima reverse transcriptase, following the manufacturer’s protocol (Fermentas).

The qRT-PCR for analysis of transcripts of Nor-1, Nurr-1 and Nur-77 was carried out in triplicate in 20 μl reaction mixture containing Maxima SYBR green master mix (Fermentas), synthesized complementary DNA and gene specific primers (1 nM) on a Stratagene Mx3005P (Agilent Technologies Inc.). Table 1 lists the primers used.

**Table 1:** Primer sequences used in quantitative RT-PCR

Gene	Primer	Annealing temperature
Nor-1	F 5′-CTCTAAAGACGGAACCGCCA-3′ R 5′-ATGAGGGCCTGGAGGGTATC-3′	60 °C
Nurr-1	F 5′-CTATTCCAGGTTCCAGGCGA-3′ R 5′-CATTCCCCAAAGCCACGAAC-3′	60 °C
Nur-77	F 5′-CACGGAGCGCTTAAGAGGAG-3′ R 5′-AGGCAGGTCTTGGCAACAAT-3′	60 °C
18S	F 5′-GGAGAGGGAGCCTGAGAAAC-3′ R 5′-CCTCCAATGGATCCTCGTTA-3’	60 °C

The temperature profiles for the amplification of target sequences were: initial denaturation at 95 °C for 10 min, followed by 40 cycles at 95 °C for 15 s, amplification for 1 min at primer-specific annealing temperatures, and then a final melting curve analysis with a range from 60° to 95 °C over 20 min. A single peak in the dissociation curve confirmed the gene-specific amplification. 18S rRNA was run in parallel to the genes of interest to normalize the average threshold cycle (Ct) values. The fold change in gene expression was calculated using ΔΔCt analysis.

### Western blotting

Cells (0.1 × 10^6^/well) were seeded in 6-well culture plates, left overnight, and then starved of FBS for at least 4 h before the addition of forskolin (25 μM), hCG (5 IU/ml) or GnRH (10 ng/ml) in Ham’s F-12 medium supplemented with 1 × ITS + 1. Incubation was for 0, 2 or 48 h, whereupon the medium was removed and cells were lysed in 100 μl of lysis buffer consisting of 20 mM Tris-HCl, 10% glycerol, 0.2 mM EDTA, 0.137 M NaCl and 1% NP-40 supplemented with a complete protease and phosphatase inhibitor cocktail (Roche Diagnostics GmbH). This was followed by 3 rapid freeze-and-thaw cycles to ensure complete cell lysis. Cell lysates were centrifuged at 12,000×g for 10 min at 4 °C and the supernatant was collected.

The amount of protein in each sample was quantified using the BCA colorimetric assay (Thermo Fisher Scientific) with (BSA) as the standard. Cell lysates (30 μg/lane) were resolved by 0.1% SDS-10% polyacrylamide gel electrophoresis (SDS–PAGE) and processed for Western blotting. After protein transfer, the nitrocellulose membrane was blocked in 5% BSA in TBST (50 mM Tris-HCl, 150 mM NaCl, 0.1% Tween-20; pH -7.4) and further incubated at 4 °C overnight with an optimized dilution of 1:1000 of rabbit polyclonal antibodies against Nor-1, Nur-77 (Thermo Fisher Scientific) and GAPDH (Cell Signaling Technology Inc.), and mouse monoclonal antibody against Nurr-1 (Thermo Fisher Scientific) in TBST containing 2.5% BSA. After subsequent washings with TBST, the membrane was further incubated with 1:5500 dilution of HRP-conjugated anti-rabbit or anti-mouse IgG antibody (Thermo Fisher Scientific) in TBST containing 0.1% BSA for 1 h at room temperature.

Membranes were washed three times with TBST (0.3% Tween-20) and developed using Immobilon chemiluminescent substrate (Millipore Corp.). Pictures of the chemiluminescent blots were taken with the FluorChem E system (ProteinSimple). The intensity of the bands on the Western blots was quantified using ImageJ software (http://rsb.info.nih.gov/ij/).

### Statistical analysis

Statistical analyses were performed using one-way ANOVA, and *p* ≤ 0.05 was considered statistically significant. Values are represented as means ± standard error of the mean (s.e.m.) of at least three different experiments.

## Results

### Expression prolife of NR4A nuclear orphan receptors in forskolin-treated BeWo cells

**Fig. 1:**
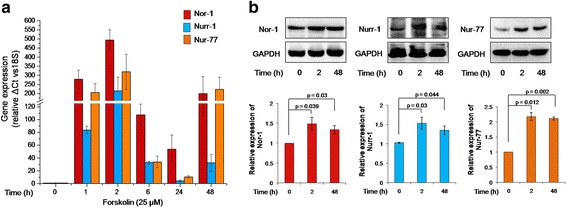
Expression profile of the members of the NR4A sub-family in BeWo cells treated with forskolin. BeWo cells were treated with forskolin (25 μM) for various time periods, followed by analysis of NR4A sub-family members at the mRNA and protein levels. **a** – Transcript profiles of Nor-1, Nurr-1 and Nur-77 in BeWo cells at 0, 1, 2, 6, 24 and 48 h of forskolin treatment. Relative expression was normalized with 18S rRNA. Data are expressed as means ± s.e.m. of three independent experiments performed in triplicate. **b** – Densitometeric plots of Nor-1, Nurr-1 and Nur-77 using GAPDH as a loading control. Data are represented as means ± s.e.m. of at least three experiments. Representative blots for the same are also shown. *p* ≤ 0.05 is considered statistically significant

To confirm the increase in Nor-1 transcription suggested by Kudo et al. [15] and to see its pattern of expression as a function of time along with other two members of the NR4A sub-family, Nurr-1 and Nur-77, BeWo cells were treated with forskolin (25 μM) for 1, 2, 6, 24 and 48 h and processed for qRT-PCR analysis as described in the Methods section. The expression of Nor-1 increased rapidly as early as 1 h, reaching its maximum expression at 2 h (~500-fold higher than the basal level; Fig. 1a). This was followed by a gradual decrease in the mRNA levels until 24 h. Interestingly, a significant increase of ~200-fold was observed at 48 h.

Nurr-1 and Nur-77 displayed a similar expression profile to Nor-1 in forskolin-treated BeWo cells, with Nurr-1 and Nur-77 levels peaking ~215-fold and ~320-fold, respectively, at 2 h, and then showing a second burst in expression level at 48 h, reaching ~35- and ~220-fold, respectively (Fig. 1a).

Nor-1, Nurr-1 and Nur-77 protein expression was also confirmed as significant via Western blotting at 2 and 48 h after forskolin treatment. The respective increases for Nor-1, Nurr-1 and Nur-77 at 2 h were ~1.48-, ~1.52- and ~2.17-fold, and at 48 h, were ~1.33-, ~1.35- and ~2.1-fold respectively (Fig 1b).

### The effect of Nor-1, Nurr-1 or Nur-77 silencing on forskolin-mediated BeWo cell fusion and hCG secretion

**Fig. 2:**
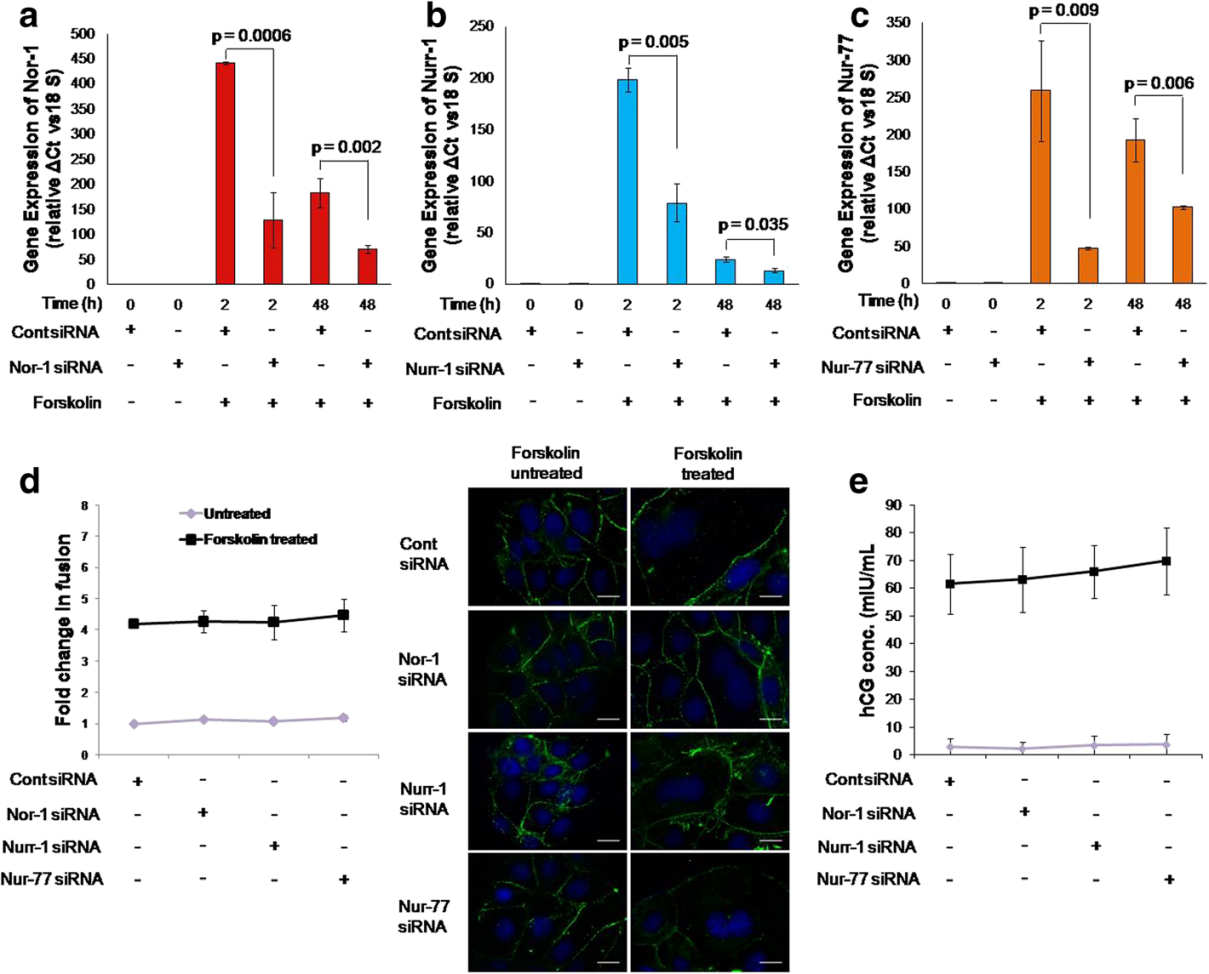
Effect of Nor-1, Nurr-1 and Nur-77 silencing on forskolin-mediated BeWo cell fusion and hCG secretion. BeWo cell knockdown for Nor-1, Nurr-1 or Nur-77 were made using siRNA as described in the Methods section. The efficacy of silencing of Nor-1, Nurr-1 or Nur-77 transcripts was confirmed via qRT-PCR using specific primers and was studied after 0, 2 and 48 h of forskolin (25 μM) treatment in control siRNA-transfected and Nor-1-, Nurr-1- or Nur-77-silenced BeWo cells. **a**, **b** and **c** – Comparisons of the transcript levels of Nor-1, Nurr-1 and Nur-77, respectively, in the control siRNA-transfected and silenced cells. Each bar represents relative expression after normalization with 18S rRNA and is expressed as means ± s.e.m. of three independent experiments performed in triplicate. The effect of Nor-1, Nurr-1 or Nur-77 silencing on forskolin-mediated BeWo cell fusion was studied at 48 h using desmoplakin I + II staining and hCG secretion was studied with ELISA. **d** – The fold change in forskolin-mediated BeWo cell fusion in Nor-1-, Nurr-1- or Nur-77-silenced cells and control siRNA-transfected cells. Values are shown as the means ± s.e.m. of three independent experiments. Representative images of desmoplakin I + II staining (green) are appended alongside. The scale bar represents 20 μm. **e** – hCG secreted by control and Nor-1-, Nurr-1- or Nur-77-silenced cells in response to forskolin treatment at 48 h and represented as means ± s.e.m. of three independent experiments performed in duplicate. *p* ≤ 0.05 is considered statistically significant

To determine the importance of the members of the NR4A sub-family in trophoblastic cell fusion, each member was silenced individually in BeWo cells using specific siRNAs. Silencing was confirmed via qRT-PCR after 2 and 48 h of forskolin treatment. A significant downregulation of Nor-1, Nurr-1 and Nur-77 transcripts were respectively observed in the Nor-1, Nurr-1 and Nur-77 silenced cells compared to the control siRNA-transfected cells (Fig. 2a–c).

The fusion efficiency and hCG secretion of the Nor-1-, Nurr-1- or Nur-77-silenced BeWo cells were analyzed to assess their differentiation potential compared to the control cells. It was observed that silencing any one of the NR4A transcription factors had no effect on forskolin-mediated BeWo cell fusion or hCG secretion (Fig. 2d, e).

### The effect of treatment of BeWo cells with hCG and GnRH on the expression of Nor-1, Nurr-1 and Nur-77

During pregnancy, various physiological factors in the uterine microenvironment influence trophoblast differentiation. The protein hormones hCG and GnRH have been shown to play a role in the upregulation of trophoblast differentiation [5, 17, 18]. These hormones activate the cAMP/PKA pathway [7, 19, 20] leading to the expression of various transcription factors and fusion-associated proteins [7, 21]. It has been shown that Nurr-1 and Nur-77 expression is upregulated by cAMP/PKA activation [22]. As forskolin is also a cAMP activator, it was hypothesized that hCG and GnRH might also affect the expression of NR4A members.

BeWo cells were treated with 5, 10, 20, 50 and 100 ng/ml doses of GnRH, and 10 ng/ml was selected as an optimum concentration because at this dose, the transcript levels of the NR4A sub-family increased significantly, while there was only a marginally higher increase at higher concentrations (data not shown).

**Fig. 3:**
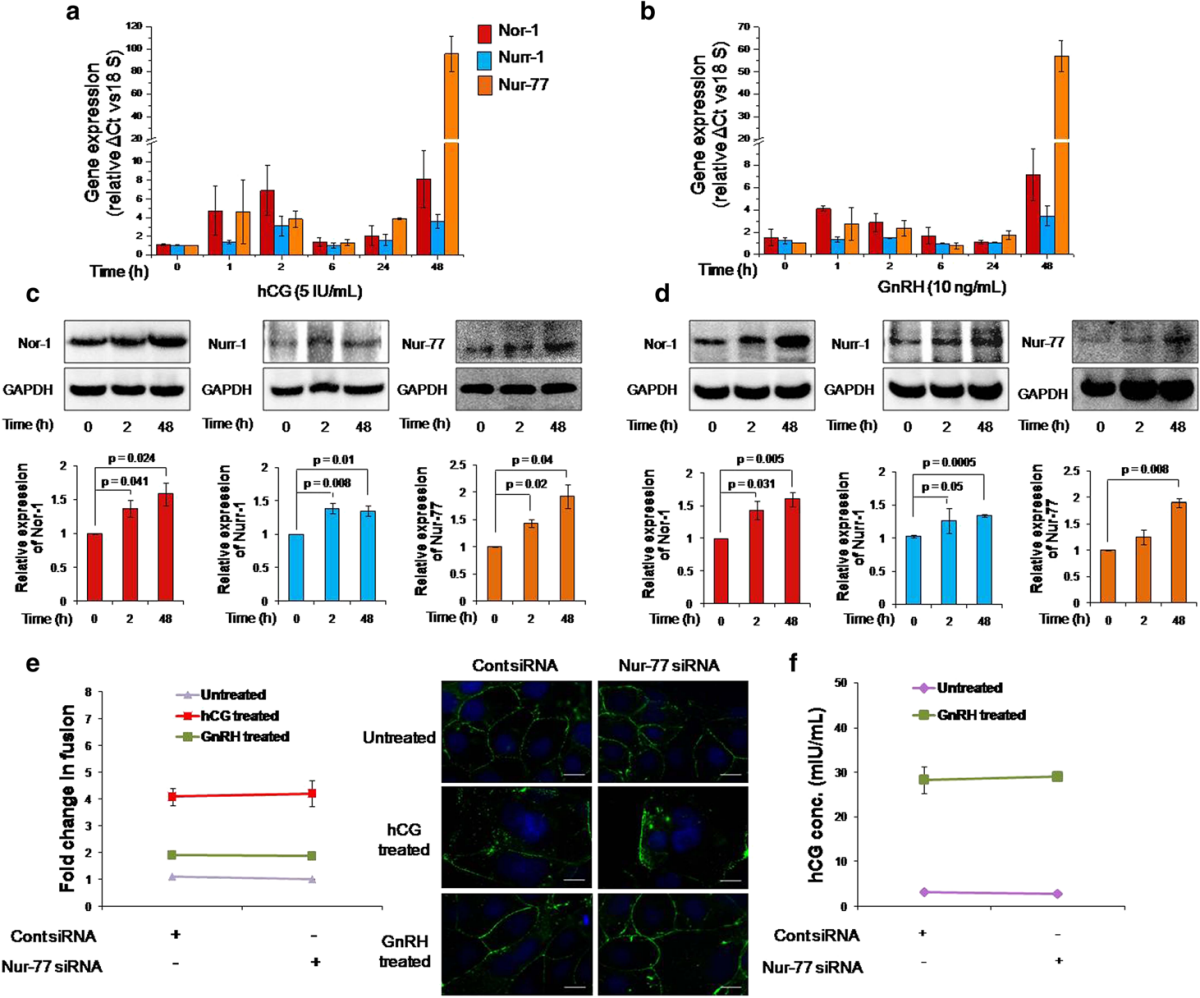
Effect of Nur-77 knockdown on hCG- and GnRH-mediated cell fusion and hCG secretion. BeWo cells were treated with either hCG (5 IU/ml) or GnRH (10 ng) for various times. Subsequently, total RNA and cell lysates were isolated and processed for qRT-PCR and Western blotting, respectively, to analyze the transcript and protein levels of Nor-1, Nurr-1 and Nur-77. **a** and **b** – qRT-PCR analysis of Nor-1, Nurr-1 and Nur-77 in the form of bar graphs of BeWo cells treated with hCG and GnRH, respectively. Relative expression was normalized with 18S rRNA. Data are expressed as means ± s.e.m. of three independent experiments performed in triplicate. **c** and **d** – Protein expression profiles of Nor-1, Nurr-1 and Nur-77 in BeWo cells after treatment with hCG and GnRH, respectively, with GAPDH used as an internal control. Values are expressed as means ± s.e.m. of the band intensity of three independent experiments. Representative blots for the same experiment are appended with the graphs. BeWo cells transfected with either Nur-77 siRNA or control siRNA were treated with either hCG (5 IU/ml) or GnRH (10 ng/ml). The fold change in fusion was estimated after 48 h using desmoplakin I + II staining and hCG secretion was analyzed in GnRH treated cells via ELISA. **e** – The effect of hCG and GnRH treatment in the control and Nur-77-silenced BeWo cells on fusion at 48 h. The data are expressed as means ± s.e.m. of three independent experiments. Representative images of desmoplakin I + II staining in *green* and DAPI in *blue* are appended alongside. The scale bar is 20 μm. **f** – hCG secreted by control and Nur-77-silenced BeWo cells in response to GnRH treatment at 48 h. Data are represented as means ± s.e.m. of three independent experiments performed in duplicate. *p* ≤ 0.05 is considered statistically significant

BeWo cells were also treated with hCG (5 IU/ml) and GnRH (10 ng/ml) for 1, 2, 6, 24 and 48 h and processed for Nor-1, Nurr-1 and Nur-77 mRNA estimation by qRT-PCR. Treatment with hCG for 2 h led to a significant increase in the transcript levels of Nor-1, Nurr-1 and Nur-77, respectively ~7-, ~3- and ~4-fold higher than in untreated cells. At 48 h, the Nor-1 and Nurr-1 transcript levels had respectively increased ~8- and ~3-fold, whereas Nur-77 showed a maximum increase of around 90-fold (Fig. 3a).

Similarly, BeWo cells treated with GnRH for 2 h also showed a significant increase in the Nor-1 and Nur-77 transcript levels. After 48 h of GnRH treatment, the transcript levels of Nor-1, Nurr-1 and Nur-77 had respectively increased ~7-, ~2- and ~60-fold (Fig. 3b).

The expression of Nor-1, Nurr-1 and Nur-77 at the protein level was assessed via Western blotting 2 and 48 h after treatment with hCG or GnRH. The respective significant increases in the protein expressions of Nor-1, Nurr-1 and Nur-77 were ~1.36-, ~1.39- and ~1.43-fold after 2 h of hCG treatment and ~1.58-, ~1.34- and ~1.92-fold after 48 h (Fig. 3c). Treatment of BeWo cells with GnRH also led to a significant upregulation of Nor-1, Nurr-1 and Nur-77 proteins: respectively ~1.6-, ~1.34- and ~1.9-fold higher than the untreated control after 48 h (Fig. 3d). However, 2 h of GnRH treatment led to a significant increase in the protein expression of only Nor-1 and Nurr-1 (~1.43- and ~1.26-fold, respectively; Fig. 3d).

### The impact of Nur-77 silencing on hCG- and GnRH-induced BeWo cell fusion and/or hCG secretion

As shown above, treatment of BeWo cells with either hCG or GnRH led to a substantial upregulation of Nur-77 expression. The next question to address was whether silencing Nur-77 would impede hCG- or GnRH-mediated differentiation of BeWo cells.

To accomplish this, Nur-77-silenced BeWo cells were treated for 48 h with either hCG (5 IU/ml) or GnRH (10 ng/ml) and assessed for cell fusion via desmoplakin I + II staining and/or hCG secretion via ELISA. However, Nur-77-silenced BeWo cells did not show any significant difference in either hCG-or GnRH-mediated BeWo cell fusion compared to the control siRNA-transfected cells at 48 h (Fig. 3e). hCG secretion in Nur-77-knockdown BeWo cells in response to GnRH treatment was comparable to that for control siRNA-transfected cells at 48 h (Fig. 3f).

### Silencing any one member of the NR4A sub-family led to a compensatory increase in the transcript levels of either one or both the other members

Although there was a significant increase in the expression of members of NR4A sub-family of nuclear orphan receptors in BeWo cells on treatment with forskolin, hCG or GnRH at early (2 h) and/or late time points (48 h), their silencing did not have an effect on BeWo cell fusion. There are few reports highlighting the functional redundancy of the two or all three members of NR4A sub-family [23, 24]. One study showed an increase in Nurr-1 expression in the adrenal glands of Nur-77-knockout mice compared to that for the wild-type counterpart [23]. Therefore, it was hypothesized that in case of BeWo cells, the compensatory increase in the expression of other members of this sub-family may be responsible for no observable phenotype (i.e., BeWo cell differentiation) in the Nor-1-, Nurr-1- or Nur-77**-**silenced cells.

**Fig. 4:**
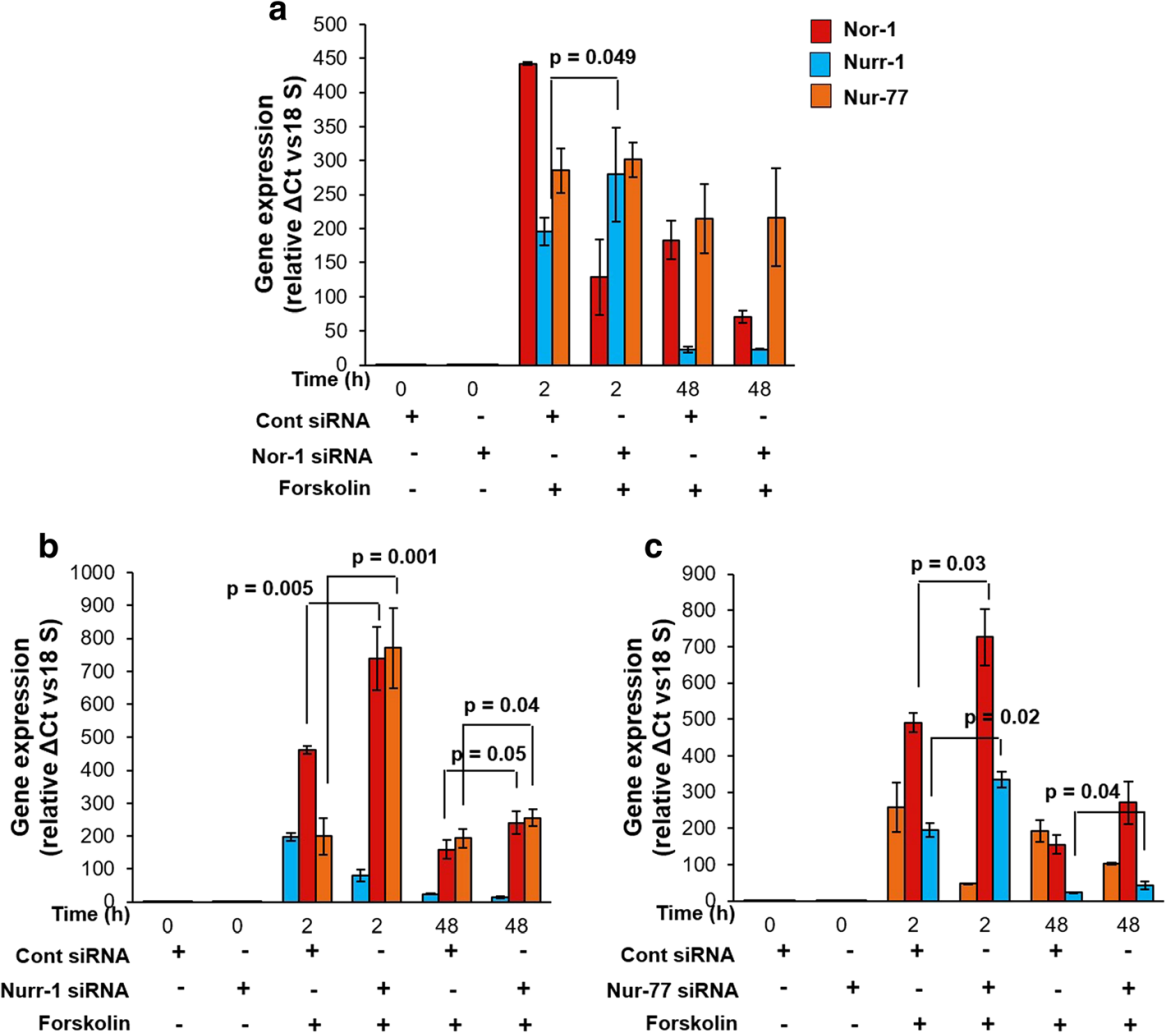
Transcript profiles of NR4A members in BeWo cells transfected with Nor-1, Nurr-1 or Nur-77 siRNA after forskolin treatment. BeWo cells were silenced for Nor-1, Nurr-1 or Nur-77 using the respective siRNA and transcript levels of all the three members were assessed using qRT-PCR after 0, 2 and 48 h of forskolin (25 μM) treatment. **a**, **b** and **c** – qRT-PCR analysis of the transcript levels of all three members in BeWo cells with Nor-1, Nurr-1 and Nur-77 knockdown, respectively. Each bar represents the relative expression after normalization with 18S rRNA, expressed as means ± s.e.m. of three independent experiments performed in triplicate. *p* ≤ 0.05 is considered statistically significant

To verify this possibility, Nor-1-, Nurr-1- or Nur-77-silenced BeWo cells treated with forskolin for 0, 2 and 48 h were subjected to qRT-PCR to evaluate the transcript levels of all three members of the NR4A sub-family. As shown in Fig. 4a, in the Nor-1-silenced cells, the expression of Nurr-1 was significantly upregulated at 2 h. In the Nurr-1 silenced cells, significant increases in transcripts of Nor-1 and Nur-77 were observed both at 2 and 48 h of forskolin treatment (Fig. 4b). Likewise, in case of Nur-77-knockdown BeWo cells, the expression of Nurr-1 was significantly increased at both 2 and 48 h, whereas Nor-1 was significantly upregulated at 2 h of forskolin treatment (Fig. 4c).

### Simultaneous silencing of Nor-1, Nurr-1 and Nur-77 led to significant decrease in forskolin-or hCG-mediated BeWo cell fusion and/or hCG secretion

**Fig. 5:**
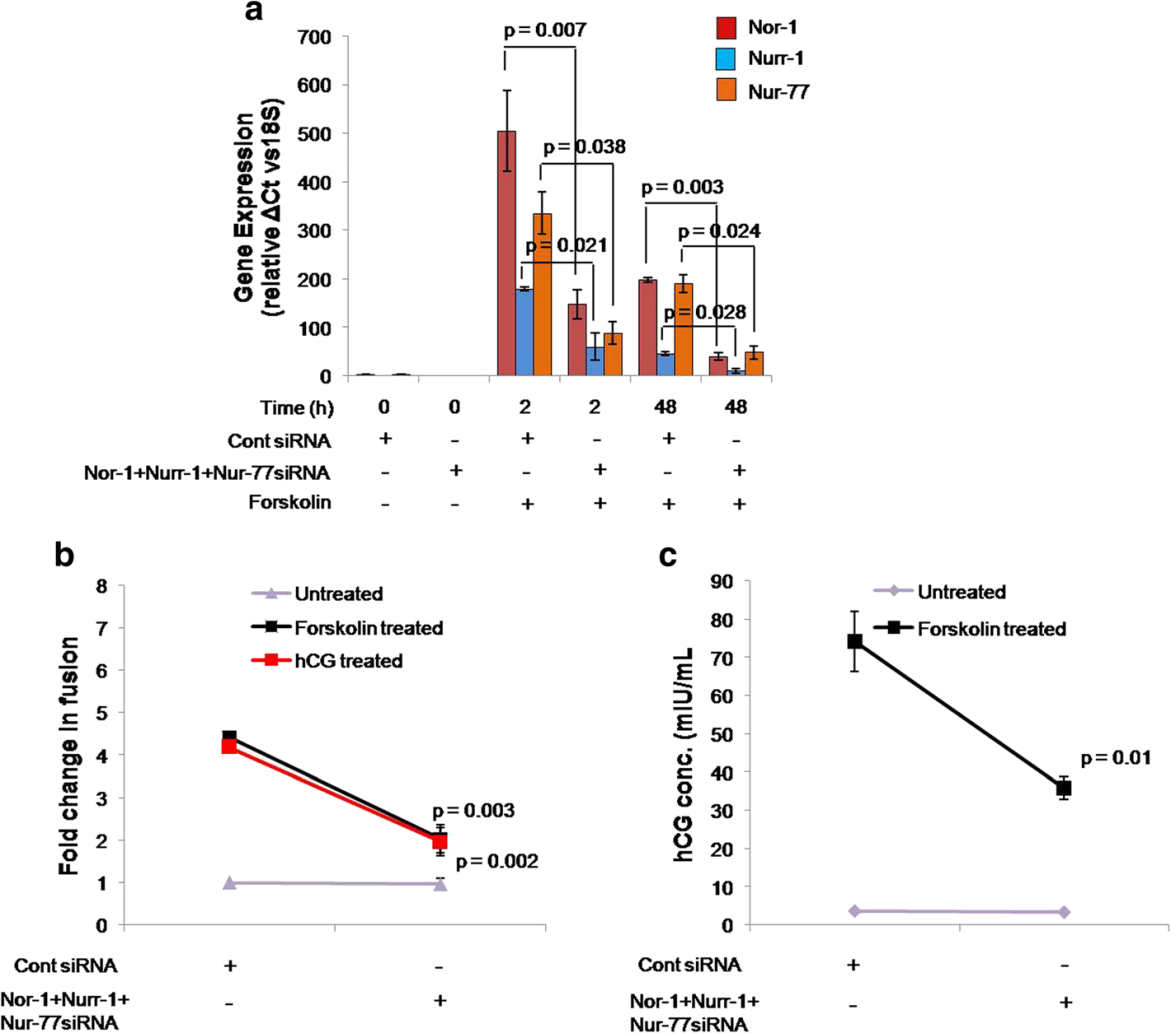
Effect of simultaneous silencing of Nor-1, Nurr-1 and Nur-77 on forskolin- or hCG-mediated BeWo cell differentiation. BeWo cells were knocked down for Nor-1, Nurr-1 and Nur-77 simultaneously using siRNA as described in Methods section. To confirm target-specific silencing of all three nuclear orphan receptors, qRT-PCR was performed at 0, 2 and 48 h after forskolin treatment in control siRNA-transfected and silenced cells. The effect of NR4A silencing on BeWo cell fusion and/or hCG secretion was studied after 48 h of forskolin and hCG treatment. **a** – Comparison of the transcript levels of Nor-1, Nurr-1 and Nur-77 in the control siRNA-transfected and NR4A-silenced cells. Each bar represents relative expression after normalization with 18S rRNA, expressed as means ± s.e.m. of three independent experiments performed in triplicate. **b** – The fold change in forskolin-mediated BeWo cell fusion in control siRNA-transfected cells and NR4A-silenced cells. Values are shown as means ± s.e.m. of three independent experiments. **c** – hCG secreted by control and NR4A-silenced cells on treatment with forskolin for 48 h. Data are represented as means ± s.e.m. of three independent experiments performed in duplicate. *p* ≤ 0.05 is considered statistically significant

To determine a conclusive role of the NR4A sub-family of nuclear orphan receptors during trophoblastic BeWo cell differentiation, all three members were silenced simultaneously. These silenced cells were then used to study forskolin- and hCG-mediated BeWo cell fusion and/or hCG secretion. Simultaneous silencing of Nor-1, Nurr-1 and Nur-77 was confirmed via qRT-PCR (Fig. 5a).

Silencing all three members of the NR4A sub-family led to a significant decrease in forskolin- and hCG-mediated BeWo cell fusion (Fig. 5b). A significant decrease in hCG secretion was also observed in BeWo cells treated with forskolin (Fig. 5c). However, this decrease in cell fusion in the NR4A silenced cells was accompanied by a substantial decrease in the number of viable cells after treatment with forskolin or hCG. Interestingly, silencing did not have any effect on cell viability when not accompanied by forskolin or hCG treatment.

## Discussion

Nuclear receptors of the NR4A sub-family are categorized as early response genes. The three members, Nur-77, Nurr-1 and Nor-1, have 90–95% homology in their DNA-binding domains but have divergent N-terminal transactivation domains. These can bind as monomers or homo-or hetero-dimers in different permutations at the nuclear receptor-binding site [13] and lead to activation of their target genes in a ligand-independent manner. This is why they are also called nuclear orphan receptors.

The basal expression of Nur-77, Nurr-1 and Nor-1was detected in various tissues but was found to be very low. However, it could be induced by a number of factors, including growth factors, peptide hormones, cytokines, fatty acids and even stress [13].

After the discovery of all three members of this sub-family, the latest being Nor-1, their functional relevance in a number of biological processes was studied, including gastrulation, neurogenesis, cell cycle regulation, apoptosis, steroidogenesis, bone development, skeletal muscle development and inflammation [13]. The microarray of BeWo cells treated with forskolin showed Nor-1 to be the highly upregulated gene [15]. Levels of Nur-77 were found to be reduced in pre-eclamptic placentae as compared with placentae from normal pregnancy [16]. In human ovaries, Nor-1 and Nurr-1 transcription factors were found to upregulate the expression of 3β-hydroxysteroid dehydrogenase type B2 (HSD3B2) [25], an enzyme that is prominently present in the placenta and metabolizes the synthesis of progesterone. Thus, the NR4A sub-family may also be associated with functional differentiation of syncytiotrophoblast through the regulation of HSD3B2 expression.

Peroxisome proliferator-activated receptor γ (PPARγ), a nuclear receptor, was also found to be a target of Nor-1 in activated alternative M2 macrophages [26]. PPARγ has been associated with an increase in hCG and syncytin-1 expression, leading to increased BeWo cell fusion. Placentae from pregnancies complicated with pre-eclampsia and IUGR show a reduced expression of PPARγ compared to placentae from normal pregnancy [27].

The expression of NR4A members is known to be responsive to cAMP second messenger pathways [22, 28, 29]. In smooth muscles, LDL (low density lipoprotein) induces Nor-1 expression by activation of PKA, MAPK (p44/p42 & p38) and CREB [22]. A study also reported a CREB-1-binding site in the promoter region of Nurr-1 [30]. Furthermore, cAMP/PKA signaling serves as a primary pathway involved in Nur-77 activation in response to GnRH in the mouse pituitary cell line [31]. Various signaling pathways and transcription factors are known to be involved during trophoblast and BeWo cell differentiation, including cAMP/PKA signaling, MAPK (p44/p42 and p38) signaling and CREB activation [4, 7]. In BeWo cells, NR4A receptor expression could be mediated via cAMP downstream pathways and CREB activation.

To assess a possible role for the three members of the NR4A sub-family during trophoblastic cell fusion, BeWo cells were treated with forskolin and their expressions were observed as a function of time. The transcript profiles of Nor-1, Nurr-1 and Nur-77 increased as early as 1 h after treatment with forskolin, peaking at 2 h and thereafter decreasing until 24 h. Interestingly, an increase in their levels was observed at 48 h (Fig. 1a). The expressions of Nor-1, Nurr-1 and Nur-77 at the protein level were also significantly upregulated after 2 and/or 48 h of forskolin treatment (Fig. 1b).

hCG is known to upregulate trophoblastic cell fusion in an autocrine manner [5, 7]. GnRH, a decapeptide hormone produced by the hypothalamus, has a major role in stimulating the pituitary hormones LH (luteinizing hormone) and FSH (follicle-stimulating hormone). It has also been shown to have extra-pituitary functions, prominently in the placental trophoblasts [32–35]. The expression of GnRH and its receptor has also been observed in the placenta in the first trimester, in the cytotrophoblasts and in the syncytiotrophoblasts, suggesting an autocrine role during trophoblast development [18]. GnRH has also been known to induce the secretion of hCG from cultured trophoblasts in a receptor-mediated fashion [17] and has also been shown to upregulate Nur-77 in the mouse gonadotroph cell line LβT2 in a PKA-dependent manner [28]. Therefore, along with forskolin, hCG and GnRH were also used to treat BeWo cells and observe their effect on the expression of NR4A receptors as a function of time. Although an increase in Nor-1 and Nurr-1 expression was observed at 2 h and/or 48 h, Nur-77 showed a substantial increase at 48 h after treatment with hCG or GnRH (Fig. 3a, b).

To determine the role of NR4A sub-family members in trophoblastic BeWo cell differentiation and fusion, their expression was individually silenced in BeWo cells using siRNA for Nor-1, Nurr-1 and Nur-77, and fusion was assessed after forskolin treatment. However, no significant difference in BeWo cell fusion or hCG secretion was observed on silencing any one of these members (Fig. 2d, e). As Nur-77 was highly upregulated in BeWo cells treated with hCG and GnRH, its expression was silenced in BeWo cells and fusion was assessed after treatment with hCG and GnRH. As observed with forskolin, silencing Nur-77 had no impact on hCG- or GnRH-induced fusion of BeWo cells or hCG secretion in the presence of GnRH (Fig. 3e, f).

Functional redundancy has been observed amongst the members of the NR4A sub-family. In the case of T cells, Nur-77 was associated with T-cell receptor-induced apoptosis, but Nur-77-deficient mice did not have an observable phenotype [24]. It was found that the other two members of the NR4A sub-family can also bind to the same DNA site, so these may be able to take over Nur-77 function in T cells. As Nurr-1 was undetectable in T cells, Nor-1 was assumed to carry out the above effect. Using a dominant negative Nur-77 protein, complete abrogation of T cell apoptosis was observed [24].

Despite the functional role of Nur-77 in leydig cells, in another study, Nur-77-deficient mice showed no apparent reproductive deficiency of the compromised phenotype. However, these mice were shown to overexpress Nurr-1 in the adrenal glands. Unfortunately, that study failed to check Nurr-1 expression in the leydig cells [36].

On similar grounds, the expressions of Nurr-1 and Nur-77 were tested in Nor-1-silenced BeWo cells and compared to control siRNA-transfected cells after treatment with forskolin. It was observed that in case of Nor-1-silenced cells, Nurr-1 expression increased significantly after 2 h of forskolin treatment (Fig. 4a). However, the levels of Nurr-1 and Nur-77 after 48 h of forskolin treatment were comparable with the control siRNA-transfected cells.

In Nurr-1-knockdown cells, both Nor-1 and Nur-77 were found to be overexpressed compared with the control siRNA-transfected cells after 2 and 48 h of forskolin treatment (Fig. 4b). Likewise, in Nur-77-silenced cells, the expressions of Nor-1 and Nurr-1 were higher than for the control cells (Fig. 4c).

With Nor-1 silencing, the expression of the other two members was not drastically upregulated, but with Nurr-1 and Nur-77 silencing, there was significant overexpression of the other two sub-family members. This might indicate their redundant role in the case of trophoblastic cell differentiation. In the case of Nor-1 knockdown, it could be that the observed expressions of Nurr-1 and Nur-77 after treatment with forskolin are adequate to compensate for Nor-1 deficiency and therefore do not require a drastic increase.

With this in mind, at best we can assume a supportive role for the NR4A sub-family in trophoblast differentiation, because their expression is upregulated in BeWo cells in response to fusion-promoting factors. However, more direct evidence is needed to determine their relevance during this process.

To gain this evidence, all three members of NR4A sub-family were silenced simultaneously for a better understanding of their involvement in BeWo cell fusion mediated by forskolin or hCG. Quantitative RT-PCR results for cells with simultaneous silencing of Nor-1, Nurr-1 and Nur-77 were compared to those for the control siRNA-transfected cells after 2 and 48 h of forskolin treatment (Fig. 5a). Silencing all three members of the NR4A sub-family led to a significant decrease in forskolin- and hCG-mediated BeWo cell fusion (Fig. 5b). A significant decrease in hCG secretion was also observed in BeWo cells treated with forskolin (Fig. 5c). However, this decrease in cell fusion in the NR4A-silenced cells was accompanied by a substantial decrease in the number of viable cells on treatment with forskolin or hCG as compared to the control siRNA-transfected cells under similar conditions. Interestingly, NR4A-silenced cells not treated with forskolin or hCG displayed no impact on cell viability when compared with the control siRNA-transfected cells.

It is known that hCG induces endoplasmic reticulum stress, triggers apoptosis and reduces steroidogenic enzyme expression in the Leydig cells of the testis [37]. Similarly, forskolin is known to induce endoplasmic reticulum and oxidative stress, triggering apoptosis [38, 39]. In mouse neurons, members of the NR4A sub-family were shown to be involved in cell survival against excitotoxic and oxidative stress by regulating the expression of neuroprotective genes. It was also found that mice with null mutations in three of six NR4A alleles showed increased neuronal damage [40]. Therefore, in the case of BeWo cells, it is possible that members of the NR4A sub-family may also be involved in cell survival through protective action against various cellular stresses, including forskolin and hCG stimulation.

Summarizing these results, it could be suggested that nuclear orphan receptors of the NR4A sub-family could be associated with trophoblast cell differentiation. Pharmacological and physiological inducers of BeWo cell differentiation like forskolin, hCG and GnRH were found to upregulate the transcript and the protein expression of Nor-1, Nurr-1 and Nur-77. Although individually silencing each member of this sub-family did not affect BeWo cell differentiation, it was accompanied with an increase in the transcript levels of other NR4A sub-family members, showing a compensatory effect, possibly due to their redundant roles. However, on silencing all three members simultaneously, a decrease in forskolin- and hCG-mediated BeWo cell fusion was observed. Knocking down the expression of the NR4A sub-family members also led to a substantial amount of cell death in response to forskolin or hCG treatment. This indicates an additional role of the NR4A sub-family in cell survival against stress. A better representation of their role could be obtained using a conditional triple knockout or the dominant negative Nor-1/Nurr-1/Nur-77 protein. This would help in enriching the existing knowledge with respect to the regulation of trophoblastic cell differentiation.

## Abbreviations

BSA: Bovine serum albumin
cAMP: Cyclic adenosine monophosphate
FBS: Fetal bovine serum
GnRH: Gonadotropin-releasing hormone
hCG: Human chorionic gonadotropin
Nor-1: neuron-derived orphan receptor 1
NR4A: Nuclear receptor sub-family 4 group A
Nur-77: Neuron-derived clone 77
Nurr-1: Nuclear receptor-related protein-1

## References

[1] Red-Horse K, Zhou Y, Genbacev O, Prakobphol A, Foulk R, McMaster M, Fisher SJ (2004). Trophoblast differentiation during embryo implantation and formation of the maternal-fetal interface. J Clin Invest.

[2] Langbein M, Strick R, Strissel PL, Vogt N, Parsch H, Beckmann MW, Schild RL (2008). Impaired cytotrophoblast cell-cell fusion is associated with reduced syncytin and increased apoptosis in patients with placental dysfunction. Mol Reprod Dev.

[3] Gauster M, Moser G, Orendi K, Huppertz B (2009). Factors involved in regulating trophoblast fusion: potential role in the development of preeclampsia. Placenta.

[4] Gupta SK, Malhotra SS, Malik A, Verma S, Chaudhary P (2016). Cell signaling pathways involved during invasion and syncytialization of trophoblast cells. Am J Reprod Immunol.

[5] Shi QJ, Lei ZM, Rao CV, Lin J (1993). Novel role of human chorionic gonadotropin in differentiation of human cytotrophoblasts. Endocrinology.

[6] Yang M, Lei ZM, Rao CV (2003). The central role of human chorionic gonadotropin in the formation of human placental syncytium. Endocrinology.

[7] Malhotra SS, Suman P, Gupta SK (2015). Alpha or beta human chorionic gonadotropin knockdown decrease BeWo cell fusion by down-regulating PKA and CREB activation. Sci Rep.

[8] Drewlo S, Baczyk D, Dunk C, Kingdom J (2008). Fusion assays and models for the trophoblast. Methods Mol Biol.

[9] Keryer G, Alsat E, Tasken K, Evain-Brion D (1998). Cyclic AMP-dependent protein kinases and human trophoblast cell differentiation in vitro. J Cell Sci.

[10] Handwerger S (2010). New insights into the regulation of human cytotrophoblast cell differentiation. Mol Cell Endocrinol.

[11] Wang Z, Benoit G, Liu J, Prasad S, Aarnisalo P, Liu X, Xu H, Walker NP, Perlmann T (2003). Structure and function of Nurr1 identifies a class of ligand-independent nuclear receptors. Nature.

[12] Baker KD, Shewchuk LM, Kozlova T, Makishima M, Hassell A, Wisely B, Caravella JA, Lambert MH, Reinking JL, Krause H (2003). The drosophila orphan nuclear receptor DHR38 mediates an atypical ecdysteroid signaling pathway. Cell.

[13] Kurakula K, Koenis DS, van Tiel CM, de Vries CJ (2014). NR4A nuclear receptors are orphans but not lonesome. Biochim Biophys Acta.

[14] Philips A, Lesage S, Gingras R, Maira MH, Gauthier Y, Hugo P, Drouin J (1997). Novel dimeric Nur77 signaling mechanism in endocrine and lymphoid cells. Mol Cell Biol.

[15] Kudo Y, Boyd CA, Sargent IL, Redman CW, Lee JM, Freeman TC (2004). An analysis using DNA microarray of the time course of gene expression during syncytialization of a human placental cell line (BeWo). Placenta.

[16] Zhang X, Yan G, Diao Z, Sun H, Hu Y (2012). NUR77 inhibits the expression of TIMP2 and increases the migration and invasion of HTR-8/SVneo cells induced by CYR61. Placenta.

[17] Barnea ER, Kaplan M, Naor Z (1991). Comparative stimulatory effect of gonadotrophin releasing hormone (GnRH) and GnRH agonist upon pulsatile human chorionic gonadotrophin secretion in superfused placental explants: reversible inhibition by a GnRH antagonist. Hum Reprod.

[18] Wolfahrt S, Kleine B, Rossmanith WG (1998). Detection of gonadotrophin releasing hormone and its receptor mRNA in human placental trophoblasts using in-situ reverse transcription-polymerase chain reaction. Mol Hum Reprod.

[19] Grafer CM, Thomas R, Lambrakos L, Montoya I, White S, Halvorson LM (2009). GnRH stimulates expression of PACAP in the pituitary gonadotropes via both the PKA and PKC signaling systems. Mol Endocrinol.

[20] Mutiara S, Kanasaki H, Oride A, Purwana IN, Shimasaki S, Yamamoto H, Miyazaki K (2009). Follistatin gene expression by gonadotropin-releasing hormone: a role for cyclic AMP and mitogen-activated protein kinase signaling pathways in clonal gonadotroph LbetaT2 cells. Mol Cell Endocrinol.

[21] Weedon-Fekjær MS, Taskén K (2012). Review: spatiotemporal dynamics of hCG/cAMP signaling and regulation of placental function. Placenta.

[22] Kovalovsky D, Refojo D, Liberman AC, Hochbaum D, Pereda MP, Coso OA, Stalla GK, Holsboer F, Arzt E (2002). Activation and induction of NUR77/NURR1 in corticotrophs by CRH/cAMP: involvement of calcium, protein kinase a, and MAPK pathways. Mol Endocrinol.

[23] Crawford PA, Sadovsky Y, Woodson K, Lee SL, Milbrandt J (1995). Adrenocortical function and regulation of the steroid 21-hydroxylase gene in NGFI-B-deficient mice. Mol Cell Biol.

[24] Cheng LE, Chan FK, Cado D, Winoto A (1997). Functional redundancy of the Nur77 and Nor-1 orphan steroid receptors in T-cell apoptosis. EMBO J.

[25] Havelock JC, Smith AL, Seely JB, Dooley CA, Rodgers RJ, Rainey WE, Carr BR (2005). The NGFI-B family of transcription factors regulates expression of 3beta-hydroxysteroid dehydrogenase type 2 in the human ovary. Mol Hum Reprod.

[26] De Paoli F, Eeckhoute J, Copin C, Vanhoutte J, Duhem C, Derudas B, Dubois-Chevalier J, Colin S, Zawadzki C, Jude B (2015). The neuron-derived orphan receptor 1 (NOR1) is induced upon human alternative macrophage polarization and stimulates the expression of markers of the M2 phenotype. Atherosclerosis.

[27] Ruebner M, Langbein M, Strissel PL, Henke C, Schmidt D, Goecke TW, Faschingbauer F, Schild RL, Beckmann MW, Strick R (2012). Regulation of the human endogenous retroviral Syncytin-1 and cell-cell fusion by the nuclear hormone receptors PPARγ/RXRα in placentogenesis. J Cell Biochem.

[28] Hamid T, Malik MT, Millar RP, Kakar SS (2008). Protein kinase a serves as a primary pathway in activation of Nur77 expression by gonadotropin-releasing hormone in the LbetaT2 mouse pituitary gonadotroph tumor cell line. Int J Oncol.

[29] Maxwell MA, Cleasby ME, Harding A, Stark A, Cooney GJ, Muscat GE (2005). Nur77 regulates lipolysis in skeletal muscle cells. Evidence for cross-talk between the beta-adrenergic and an orphan nuclear hormone receptor pathway. J Biol Chem.

[30] Rius J, Martínez-González J, Crespo J, Badimon L (2004). Involvement of neuron-derived orphan receptor-1 (NOR-1) in LDL-induced mitogenic stimulus in vascular smooth muscle cells: role of CREB. Arterioscler Thromb Vasc Biol.

[31] McEvoy AN, Murphy EA, Ponnio T, Conneely OM, Bresnihan B, FitzGerald O, Murphy EP (2002). Activation of nuclear orphan receptor NURR1 transcription by NF-kappa B and cyclic adenosine 5′-monophosphate response element-binding protein in rheumatoid arthritis synovial tissue. J Immunol.

[32] Khodr GS, Siler-Khodr T (1978). Localization of luteinizing hormone-releasing factor in the human placenta. Fertil Steril.

[33] Siler-Khodr TM, Khodr GS (1981). Dose response analysis of GnRH stimulation of hCG release from human term placenta. Biol Reprod.

[34] Kakar SS, Jennes L (1995). Expression of gonadotropin-releasing hormone and gonadotropin-releasing hormone receptor mRNAs in various non-reproductive human tissues. Cancer Lett.

[35] Lin LS, Roberts VJ, Yen SS (1995). Expression of human gonadotropin-releasing hormone receptor gene in the placenta and its functional relationship to human chorionic gonadotropin secretion. J Clin Endocrinol Metab.

[36] Martin LJ, Tremblay JJ (2010). Nuclear receptors in Leydig cell gene expression and function. Biol Reprod.

[37] Park SJ, Kim TS, Park CK, Lee SH, Kim JM, Lee KS, Lee IK, Park JW, Lawson MA, Lee DS (2013). hCG-induced endoplasmic reticulum stress triggers apoptosis and reduces steroidogenic enzyme expression through activating transcription factor 6 in Leydig cells of the testis. J Mol Endocrinol.

[38] Seo HY, Kim MK, Min AK, Kim HS, Ryu SY, Kim NK, Lee KM, Kim HJ, Choi HS, Lee KU (2010). Endoplasmic reticulum stress-induced activation of activating transcription factor 6 decreases cAMP-stimulated hepatic gluconeogenesis via inhibition of CREB. Endocrinology.

[39] Ríos-Silva M, Trujillo X, Trujillo-Hernández B, Sánchez-Pastor E, Urzúa Z, Mancilla E, Huerta M (2014). Effect of chronic administration of forskolin on glycemia and oxidative stress in rats with and without experimental diabetes. Int J Med Sci.

[40] Volakakis N, Kadkhodaei B, Joodmardi E, Wallis K, Panman L, Silvaggi J, Spiegelman BM, Perlmann T (2010). NR4A orphan nuclear receptors as mediators of CREB-dependent neuroprotection. Proc Natl Acad Sci U S A.

